# Deep CNNs with Robust LBP Guiding Pooling for Face Recognition

**DOI:** 10.3390/s18113876

**Published:** 2018-11-10

**Authors:** Zhongjian Ma, Yuanyuan Ding, Baoqing Li, Xiaobing Yuan

**Affiliations:** 1 Science and Technology on Microsystem Laboratory, Shanghai Institute of Microsystem and Information Technology, Chinese Academy of Sciences, Shanghai 201800, China; mazj@mail.sim.ac.cn (Z.M.); sinoiot@mail.sim.ac.cn (B.L.); 2 University of Chinese Academy of Sciences, Beijing 100049, China; 3 International Business Machines Corporation, Shanghai 201800, China; dyuany@cn.ibm.com

**Keywords:** pooling layer, CNNs, noise, LBP, feature maps

## Abstract

Pooling layer in Convolutional Neural Networks (CNNs) is designed to reduce dimensions and computational complexity. Unfortunately, CNN is easily disturbed by noise in images when extracting features from input images. The traditional pooling layer directly samples the input feature maps without considering whether they are affected by noise, which brings about accumulated noise in the subsequent feature maps as well as undesirable network outputs. To address this issue, a robust Local Binary Pattern (LBP) Guiding Pooling (G-RLBP) mechanism is proposed in this paper to down sample the input feature maps and lower the noise impact simultaneously. The proposed G-RLBP method calculates the weighted average of all pixels in the sliding window of this pooling layer as the final results based on their corresponding probabilities of being affected by noise, thus lowers the noise impact from input images at the first several layers of the CNNs. The experimental results show that the carefully designed G-RLBP layer can successfully lower the noise impact and improve the recognition rates of the CNN models over the traditional pooling layer. The performance gain of the G-RLBP is quite remarkable when the images are severely affected by noise.

## 1. Introduction

Nowadays, using deep learning architectures to dig out information and extract features from images have drawn a lot of attention in computer vision and machine learning tasks. Among them, CNN has gradually become the most effective method since it can extract essential features quickly from images and has been widely applied in face recognition, target tracking, expression analysis, and other fields. For instance, in 2014, the Deepface [1] method came out and achieved 97.35% accuracy on the LFW database [2]. In DeepID2 [3] and DeepID2+ [4] models, the authors skillfully combined face identification and verification to increase inter-class variations and reduce intra-class variations simultaneously. This mechanism successfully gets an obvious improvement on some typical databases. DeepID3 [5] further enlarges and deepens the network, finally reaching a 99.53% accuracy on the LFW database. VGGnet [6] is another influential model to learn effective features from input images which has been used in many visual recognition tasks [7,8,9]. In this model, many convolutional layers are stacked together to get some more complex features. The GoogleNet [10] was proposed which ranked in the top in the ILSVRC 2014. There are many inception modules in this model which combine pooling with convolutional layers to form a new feature extraction layer. Moreover, in 2014, Gong et al. [11] proposed the Multi-scale Orderless Pooling (MOP) CNN to extract CNN activations for local patches at multiple scale levels. Later, faster R-CNN [12] proposed in 2016 merges the Region Proposal Network (RPN) and Fast R-CNN [13] into a single network by sharing their convolutional features which is not only a cost-efficient solution for practical usage but also an effective way to improve object detection accuracy.

**Figure 1:**
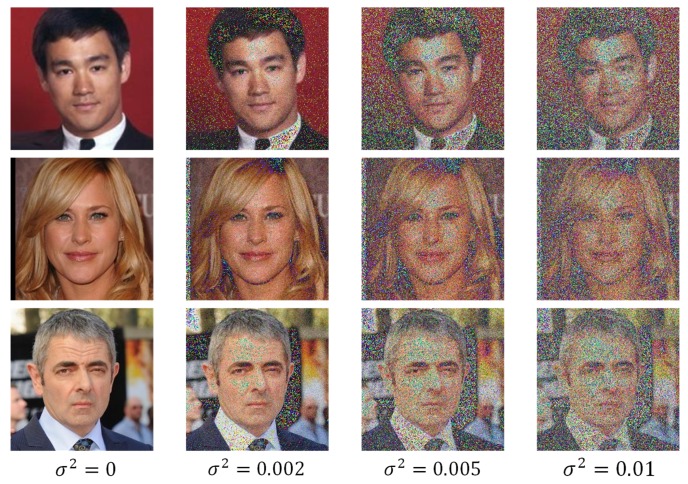
The sample testing images which were injected with different intensity of Gaussian noise.

CNN is effective for visual recognition, but sometimes it is also very susceptible to the noise injected into the input images in the real-world applications. Taking Alexnet [14] and ZF-5net [15] as examples, we select 100 subjects from the CASIA-WebFace [16] to train and test the two networks (10,900 for training and 1700 for testing). To evaluate the networks in the noisy conditions, the testing images were injected with different intensity of Gaussian noise, as shown in Figure 1. It is clear that the edges and other features information in the face images become more and more challenging to recognize along with the noise intensity.

Table 1 shows the recognition rates of the Alexnet and ZF-5net. In Table 1, we can see that the recognition rates of the networks decrease drastically along with the noise intensity. When the variance of Gaussian noise increases to 0.01, the recognition rates of both networks even drop to 50% and nearly cannot be used in practice. However, in the real-world applications, the obtained face images would be easily affected by various factors and finally contain some noise during the collection, processing, and transmission.

A carefully designed CNN is mainly comprised of three types of layers: convolutional layer, pooling layer, and fully-connected layer. In general terms, the objective of pooling is to transform the common feature representations into a new, more usable one which preserves essential information while discarding irrelevant details [17]. However, most of the pooling methods such as the max pooling and the average pooling down sample the input feature maps in the corresponding sliding window based on a constant criterion and all the pixels are treated equally in these cases. Once some of the pixels in the sliding window are affected by noise, they are still probably preserved or averaged after the pooling layer since the current pooling methods have no response to the noise injected into the input. To address this issue, a new pooling method based on robust LBP guiding in deep CNNs is proposed in this paper to deal with the noise injected into the input images, which is named as RLBP Guiding Pooling (G-RLBP).

There are many effective hand-crafted methods to extract features from images. For instance, HOG [18] constructs features by computing and counting histograms of gradient directions in local regions of images. HOG features combined with SVM classifier have been widely applied in pedestrian detection. SIFT [19] feature is a very stable local feature, which is invariant to rotation, scale scaling, and luminance change. To address the impact of makeup on automated face recognition, Chen et al. [20] proposed another useful method in which a set of feature descriptors such as Local Gradient Gabor Pattern (LGGP) [21] and Densely Sampled Local Binary Pattern (DS-LBP) are utilized to represent each patch of the face images. LBP is another representative hand-crafted feature extraction method which has been widely used in many face recognition tasks [22,23,24]. Compared with other feature extraction methods such as HOG and SIFT, LBP-based methods can extract small movements in the facial images, and they are described in a much lower dimensional feature space which benefits the real-time applications. In the proposed G-RLBP, the robust LBP algorithm is utilized to guide the pooling mechanism. The proposed G-RLBP first analyses each pixel in the sliding window, calculates their probabilities affected by noise, and gets the robust LBP (RLBP) weight maps. Then, all the pixels are weighted averaged as the final results of the current sliding window in this pooling layer according to the RLBP weight maps. Here, we utilize the fact that most of the LBP patterns in the face images belong to the uniform patterns and only a small part belongs to the non-uniform patterns [22,25]. Moreover, the non-uniform patterns are usually caused by noise injected into the images. Thus, we can utilize the pattern of the pixel to guide the pooling procession to decrease the noise injected into the feature maps. In this way, the parameters of the input feature maps can be reduced as the traditional pooling methods, and the impact of noise injected into the feature maps can also be effectively lowered simultaneously. The experimental results also show that the performance of some CNNs equipped with the G-RLBP pooling layer can be improved notably in the noisy conditions.

The remainder of this paper is organized as follows. Section 2 describes the proposed G-RLBP pooling method, and its theoretical analysis is also carried out in this section. Section 3 reports the experimental design and performance comparisons of the G-RLBP pooling method. Section 4 concludes this paper.

## 2. Proposed Method

**Figure 2:**
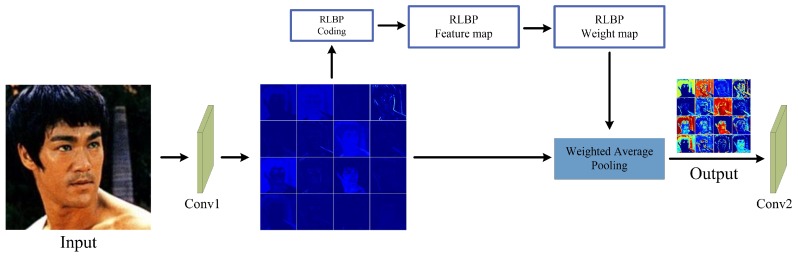
The structure of the G-RLBP pooling layer.

In the traditional CNNs, the convolutional layer, pooling layer and fully-connected layer care little about the noise injected into the input images. However, the noise impact introduced by the input images would accumulate layer by layer. When the intensity of the noise reaches a certain degree, the recognition rate of the network will drop sharply, as Table 1 shows. Therefore, it is highly necessary to lower noise interference at the first several layers of the network. Figure 2 is the structure of our designed G-RLBP pooling layer to reduce the noise impact in the first pooling layer. There are three main modules in the G-RLBP pooling layer:

- Convolutional Feature Maps are the outputs of the first convolutional layer of the network.
- RLBP Feature Maps are the robust LBP coding results of the convolutional feature maps.
- RLBP Weight Maps are the weights of each pixel in the sliding window according to the RLBP feature maps.

The values in the RLBP weight maps reflect the probability of each pixel affected by noise in the convolutional feature maps. Utilizing this weight maps to down sample the convolutional feature maps, the pixels which are more likely to be affected by noise would be assigned smaller weights to lower the noise interference to the networks. We give a detailed description of this RLBP weight maps in the next section.

### 2.1. Robust LBP

LBP is a very effective method to extract local texture features from images. In recent years, LBP and its variants have been successfully applied to various pattern recognition tasks, such as texture analysis, face detection, facial expression recognition and so on.

**Figure 3:**
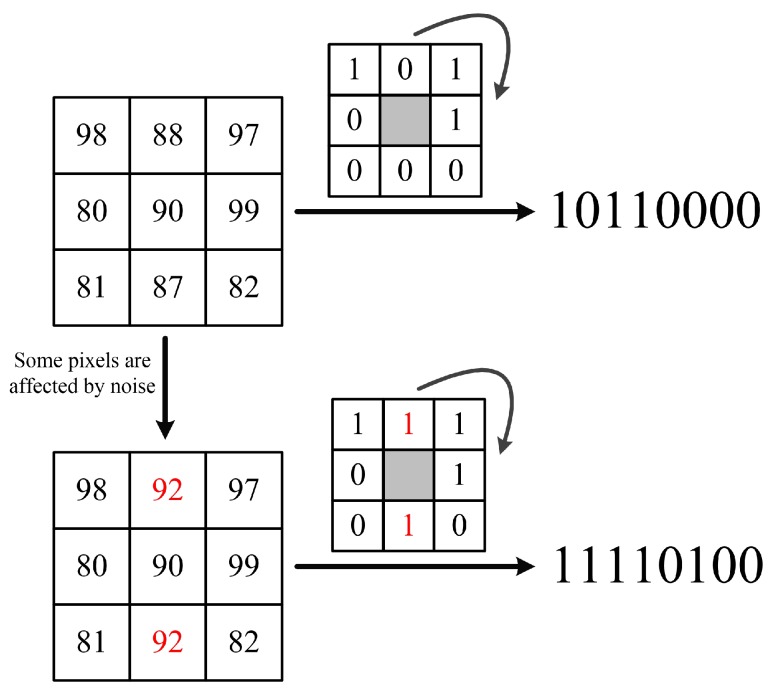
The influence of noise to the LBP coding results.

Ahonen et al. [22] firstly introduced the LBP method into face recognition field. They cut the face image into several sub-images and then calculated the LBP values of each pixel in certain sub-image. The local and overall features of the face image are combined in this method with excellent performance in real-world applications. However, in the coding process, the traditional LBP method usually compares the central pixel and its neighbors to get a binary string in the sliding window. Some small changes of the pixels finally result in very different coding results. For instance, Figure 3 shows the two coding processes of LBP; the red numbers indicate the pixels affected by noise. It is clear that the normal LBP value of the central pixel is 1011 0000; once some neighboring pixels are slightly affected by noise, the coding results would be very different from the normal one. Thus, the LBP is very sensitive to noise which can easily modify the gray pixel value of the image and may result in entirely different coding results.

In this section, we utilize a noise-robust LBP coding method called RLBP in which we can judge whether the pixel value of the image is affected by noise before feeding into the network based on its probability.

The basic LBP algorithm encodes the signs of the pixel differences between the central pixel and its neighboring pixels in a sliding window. The coding criterion is as follows:

(1) $$b\left(z_{p}\right)=\left\{\begin{array}{cc} 1, & \mathrm{if}\;\;\;z_{p}\geq 0\\ 0, & \mathrm{if}\;\;\;z_{p}<0 \end{array}\right.,$$

where $z_{p}$ indicates the difference between the central pixel and its neighbors in a sliding window (e.g., 3×3), and $z_{p}$ is encoded into 1 or 0 according to Equation (1). The central pixel and all of its neighbors are compared in turn and then these 1-bit binary numbers are connected in a certain direction to get a *P*-bits binary string (*P* is the neighbor number of the central pixel). Finally, this *P*-bits binary string is converted to a decimal number between 0 and $2^{P}$ which is regarded as the LBP value of the central pixel in this 3×3 sliding window.

**Figure 4:**
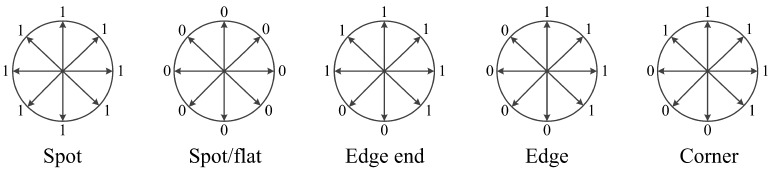
Local primitives samples of LBP uniform patterns.

There are $2^{P}$ different patterns in the LBP algorithm. Among them, P×(P−2)+2 LBP patterns are defined as uniform patterns with at most two circularly bitwise transitions from 0 to 1 or vice versa, and the rest are non-uniform patterns. Most LBP values in natural images are uniform patterns [22]. Thus, uniform patterns are statistically more significant, and their occurrence probabilities can be more reliably estimated. In contrast, non-uniform patterns are statistically insignificant, and hence noise-prone and unreliable. Figure 4 shows some of the local primitives (spots, flat region, edges ends and corners) represented by uniform LBP patterns [26].

The RLBP is different from LBP in which $z_{p}$ is encoded to a ternary pattern (0, 1 and *u*) according to Equation (2).

(2) $$b\left(z_{p}\right)=\left\{\begin{array}{cc} 1, & \mathrm{if}\;\;\;z_{p}\geq t_{p}\\ u, & \mathrm{if}\;\left|z_{p}\right|<t_{p}\\ 0, & \mathrm{if}\;\;\;z_{p}\leq -t_{p} \end{array}\right.,$$

where $t_{p}$ is the threshold, *u* is an uncertain binary number and encoded to 0 or 1 in a certain probability. Obviously, a binary string which contains uncertain *u* is unable to be encoded into a certain decimal number between 0 and $2^{P}$. LBP uniform patterns can capture the main structural information of the image while reducing the noise interference in the texture. In natural images, the frequency of the uniform patterns appears far higher than the non-uniform ones. Many experimental data show that 90.6% of the LBP patterns in the face image belong to the uniform patterns, and only a small part belongs to the non-uniform patterns. Moreover, these non-uniform patterns are often caused by noise. Here is a simple experiment to explain this phenomenon in face images. For example, injecting different intensity (*d*) of salt and pepper noise into the samples of the ORL database [27], the proportion of the non-uniform patterns increases continuously along with the intensity of noise. When *d* is 0.05, 0.1 and 0.15, the proportion of the non-uniform mode is 27.86%, 31.46% and 34.49%, respectively. Based on this observation, we can preset the value of *u* in different coding patterns.

Generally, there are three cases in total according to the number of uniform patterns.

#### 2.1.1. Case 1: Only One Pattern Belongs to the Uniform Patterns

**Figure 5:**
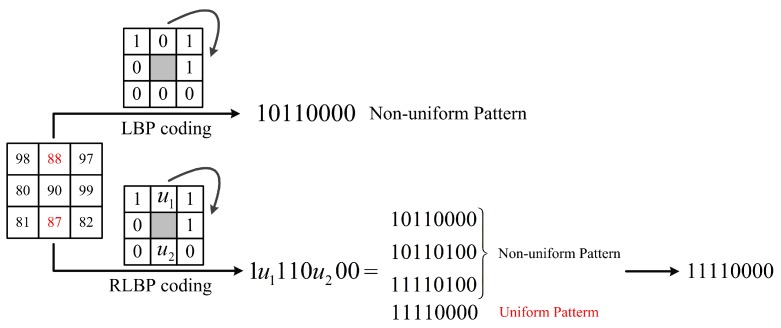
The comparison of LBP and RLBP patterns in a 3×3 sliding window with only one pattern in uncertain RLBP pattern collection belongs to the uniform patterns.

Figure 5 is a comparison of LBP and RLBP coding process in a 3×3 sliding window. Here, $t_{p}$ is set to be 5. The detailed discussion of $t_{p}$ can be seen in the Section 3.1.1 The corresponding uncertain RLBP pattern collection in the RLBP algorithm is defined as $C\left(U\right)=1u_{1}110u_{2}00$. There are two uncertain binary numbers $U=\{u_{1},u_{2}\}$. According to the combinations of ***U***, there are four different *P* = 8 bits binary strings: 1011 0000, 1011 0100, 1111 0000, and 1111 0100. Among them, 1011 0000, 1011 0100 and 1111 0100 belong to the non-uniform patterns, and they are likely to be affected by noise. Therefore, the central pixel ’90’ of the 3×3 sliding window is encoded to 1111 0000 in the RLBP algorithm because it is the only one uniform pattern and we can also calculate the probability of this code. Let p(u=1) be the probability of $z_{p}$ encoded to 1.

(3) $$p\left(u=1\right)=0.5+0.5\cdot \frac{z_{p}}{t_{p}},$$

(4) p(u=0)=1−p(u=1).

We can easily get $p(u_{1}=1)=0.3$, $p(u_{2}=0)=0.8$. Finally, the probability of the central pixel encoded to 1111 0000 is $p\left(u_{1}=1,u_{2}=0\right)=p\left(u_{1}=1\right)\cdot p\left(u_{2}=0\right)=0.24$.

#### 2.1.2. Case 2: More Than One Patterns Belong to the Uniform Patterns

**Figure 6:**
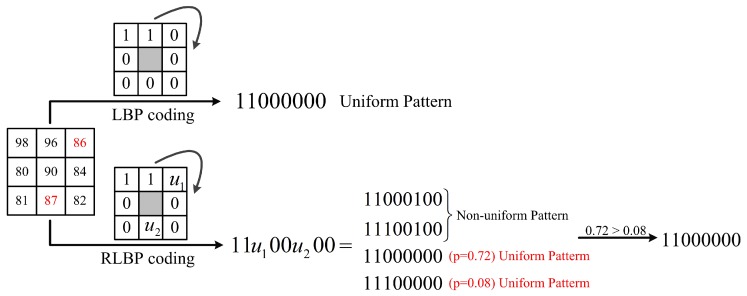
The comparison of LBP and RLBP patterns in a 3×3 sliding window with more than one patterns in uncertain RLBP pattern collection belong to the uniform patterns.

Figure 6 shows another case when more than one patterns belongs to the uniform patterns. The uncertain RLBP pattern in this case is: $11u_{1}00u_{2}00$. According to the values of $u_{1}$ and $u_{2}$, there are four candidate binary strings: 1110 0000, 1110 0100, 1100 0100, and 1100 0000. Two binary strings belong to the uniform patterns: 1110 0000 and 1100 0000. Here, according to Equations (3) and (4), we have:

(5) $$\begin{array}{c} p(u_{1}=0)=0.9,\\ p(u_{1}=1)=0.1,\\ p(u_{2}=0)=0.8,\\ p(u_{2}=1)=0.2. \end{array}$$

Then, the encoding probability of 1110 0000 and 1100 0000 are, respectively: $p\left(u_{1}=1,u_{2}=0\right)=p\left(u_{1}=1\right)\cdot p\left(u_{2}=0\right)=0.08$ and $p\left(u_{1}=0,u_{2}=0\right)=p\left(u_{1}=0\right)\cdot p\left(u_{2}=0\right)=0.72$. In the RLBP algorithm, the binary strings with the max probability is defined as the final coding result, thus the central pixel in Figure 6 is encoded as 1100 0000 with a probability of 0.72.

#### 2.1.3. Case 3: None Pattern Belongs to the Uniform Patterns

**Figure 7:**
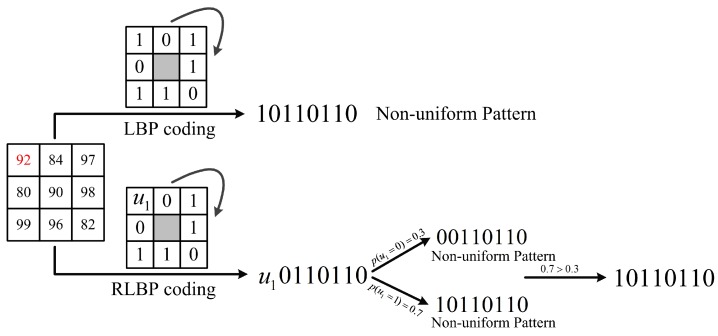
The comparison of LBP and RLBP patterns in a 3×3 sliding window with none pattern in uncertain RLBP pattern collection belongs to the uniform patterns.

In the third case, no pattern belongs to the uniform patterns, as Figure 7 shows. In Figure 7, the uncertain RLBP pattern is $C\left(U\right)=u_{1}0110110$. There are two binary strings when $u_{1}$ is set to 0 or 1: 0011 0110 and 1011 0110. They both belong to the non-uniform patterns. Therefore, the central pixel can only be encoded to 1011 0110 with a probability of $p(u_{1}=1)=0.7$.

In conclusion, the RLBP value of the central pixel in any sliding windows can be summarized as follows:

1. Calculating all the uncertain RLBP values of the central pixel according to Equation (2).
  (6) $$URLBP_{\Phi_{u}}=\left\{C\left(U\right)|U\in {\left\{0,1\right\}}^{n},C\left(U\right)\in \Phi_{u}\right\},$$
  (7) $$URLBP_{{\bar{\Phi }}_{u}}=\left\{C\left(U\right)|U\in {\left\{0,1\right\}}^{n},C\left(U\right)\in {\bar{\Phi }}_{u}\right\},$$
  where $\Phi_{u}$ and ${\bar{\Phi }}_{u}$ are the collections of the uncertain uniform patterns and non-uniform patterns. $U=\{u_{1},u_{2},\ldots ,u_{n}\}$.
2. If $URLBP_{\Phi_{u}}$ == None and $URLBP_{{\bar{\Phi }}_{u}}\neq None$, then calculate the probabilities of all non-uniform patterns in $URLBP_{{\bar{\Phi }}_{u}}$ according to Equation (7). Otherwise, calculate the probabilities of all uniform patterns in $URLBP_{\Phi_{u}}$.
  (8) $$p\left(C\left(U\right)\right)=\prod_{i=1}^{n}p(u_{i}|u_{i}\in \left\{0,1\right\}).$$
3. Finally, the pattern with the max probability is regarded as the RLBP value of the central pixel.
  (9) $$RLBP_{R,P}=arg\max_{C(U)}\left(\left\{p\left(C\left(U\right)\right)\right\}\right),$$
  where *R* indicates the radius of the current sliding window and *P* is the neighbor number of the central pixel.

### 2.2. RLBP Weight Maps

We have given a detailed discussion of the RLBP coding process. There are three cases to be considered to generate the RLBP weight maps in our G-RLBP method.

- Case 1: If the center pixel of the sliding window is encoded as only one uniform pattern according to Equation (2), the corresponding RLBP weight of the center pixel is defined as 1.
- Case 2: If the center pixel of the sliding window is encoded as more than one uniform patterns, then the probabilities of each uncertain RLBP in the $URLBP_{\Phi_{u}}$ are calculated, and the max probability is taken as the RLBP weight corresponding to the central pixel.
- Case 3: If all the uncertain RLBP patterns belong to the non-uniform, the RLBP weight of this central pixel is set to be 0.

**Figure 8:**
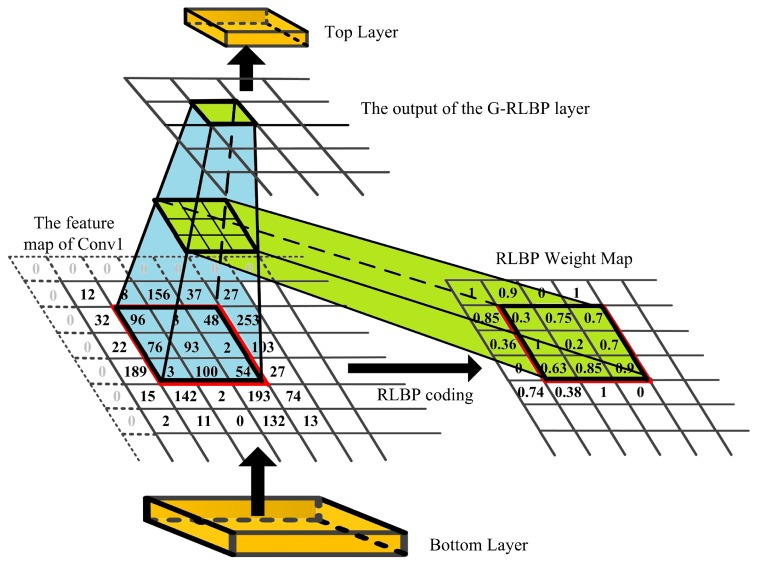
The visualization of the G-RLBP pooling process in the designed network.

Figure 8 visualizes the pooling process in our G-RLBP layer. After the RLBP pooling weight maps are generated, we can down sample the convolutional feature maps according to Equation (10):

(10) $$y=\frac{1}{N}\sum_{i=1}^{N}w_{i}\cdot x_{i},$$

where *N* is the pixel number in a sliding window, and $w_{i}$ and $x_{i}$ indicate the RLBP weight and the corresponding value in the input feature maps respectively. Here, *N* is set to 9. *y* is the output of the current sliding window after the G-RLBP pooling.

## 3. Experimental Analysis

### 3.1. Baseline Network Architectures

The Alexnet, ZF-5net, and GoogleNet are three baseline network architectures studied in the experiments. The specific configurations of the Alexnet and ZF-5net are shown in Table 2. In contrast to the Alexnet, the ZF-5net uses smaller filters in Conv1 to preserve more original pixels information. In our models, the Pool1 layers of the three baseline networks are replaced by the proposed G-RLBP layers. One point needs to be emphasized: only the Pool1 layer is replaced by the proposed G-RLBP layer. Most of the noise in the feature maps could be reduced after the first G-RLBP layer in the network which would be verified in the following sections. It fails to bring too much good effect when other pooling layers are all replaced. Besides, the G-RLBP layers need to calculate the weight maps of each pooling window, which is time-consuming to some extent. In particular, to further evaluate the proposed G-RLBP layer, we also used data augmentation by injecting different random noise into the training data as another experiment. The three networks are transferred to face recognition task through fine-tuning. The networks are implemented by Caffe toolbox [28]. Stochastic Gradient Descent (SGD) is used for optimizing in our model with back propagation. We set the weight decay and momentum to 0.005 and 0.9, respectively. The base learning rate is initially set to be 0.001 for training the original ZF-5net and Alexnet models. All networks in our experiments are trained 80 epochs. The evaluation is performed on a machine with 64G memory Xeon CPU 2.1GHz and GPU GeForce GTX1080Ti. The training database used in the experiments is the CASIA-WebFace database including 10,575 subjects with 494,414 face images which are collected from the website. We selected 100 subjects from the CASIA-WebFace database to train the models. The testing data were selected from the ORL and AR database [29]. The output of the fc6 layer is regarded as the feature extracted from the networks with a feature dimension of 4096.

In the recognition stage, the nearest neighbor classifier is introduced to calculate the distance between two feature vectors with three different distance measures: the Chi-Square distance, the Euclidean distance, and the Cosine distance. During the experiment, the AR and ORL databases were injected with different intensity of Gaussian noise and salt and pepper noise to test the performance of the G-RLBP pooling method.

#### 3.1.1. The Discussion of $t_{p}$

**Figure 9:**
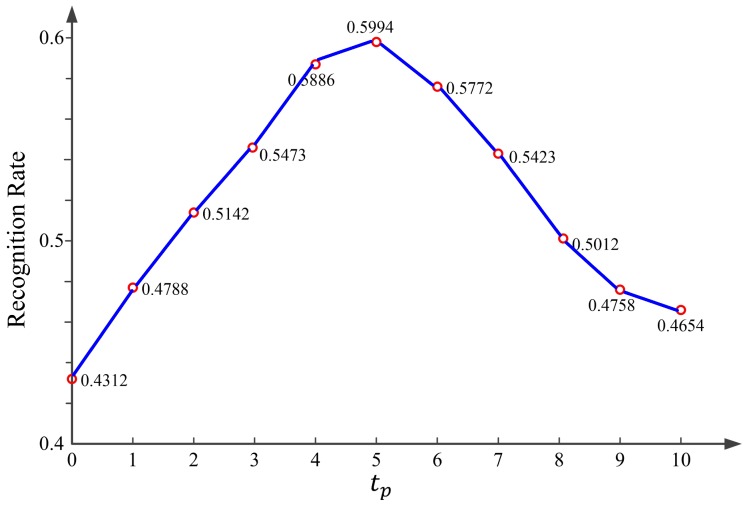
Recognition rates of the RLBP algorithm versus $t_{p}$.

$t_{p}$ is one of the important parameters in our method which affect the algorithm complexity and performance. We conducted an experiment on the ORL database to give a simple discussion of it. The histogram of the RLBP patterns was regarded as the feature of each face image. For brevity, only Euclidean distance was utilized to measure the similarity of two features. In this experiment, one image was selected as the training set and the rest as the testing set for evaluation. Figure 9 shows the recognition rates of the RLBP with respect to $t_{p}$.

We can see clearly in Figure 9 that, when $t_{p}$ is set to 5, the RLBP can get the highest recognition rate on the ORL database. Thus, in the following experiments, $t_{p}$ was set to 5, which is suitable in most of the cases.

### 3.2. Experiments on the ORL Database

The first set of experiments were carried out on the ORL database which contains 40 subjects with 10 images for each subject. One image of each subject was selected as the training set and the rest as the testing set per turn. The experiment was repeated 10 times so that each image could be used as the training set for evaluation. Before the testing images were fed into the network, we injected different intensity of Gaussian noise and salt and pepper noise to evaluate the performance of the G-RLBP pooling layer.

**Figure 10:**
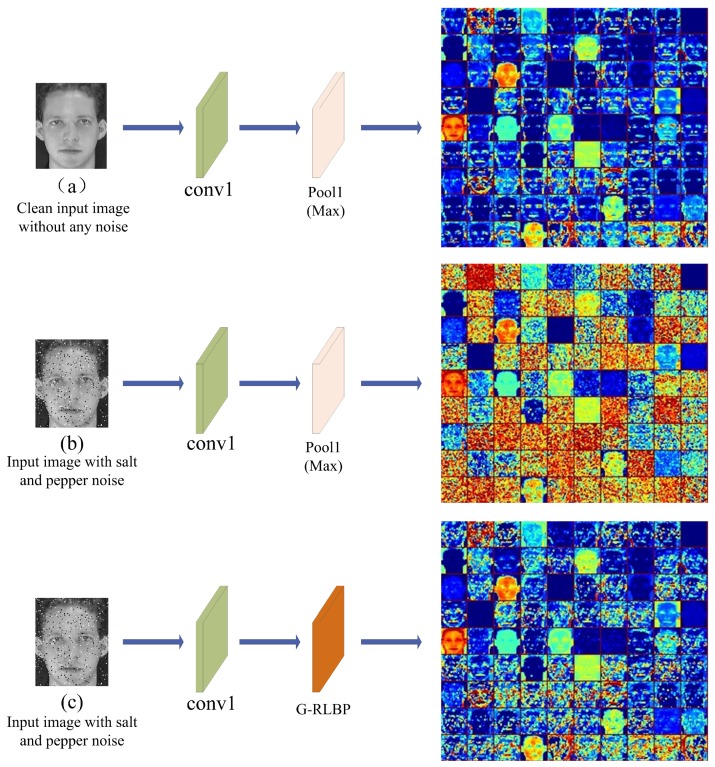
The feature maps of different pooling layers in the Alexnet. Conv1 indicates the first convolutional layer in the networks. (**a**) The output of Pool1 layer without injecting any noise into the input image in the baseline Alexnet; (**b**) the output of Pool1 layer in the baseline Alexnet when the input image is injected with salt and pepper noise (d=0.05); and (**c**) the output of the G-RLBP pooling layer when the input image is injected with the same noise as (**b**).

Figure 10 visualizes the output of the Pool1 and G-RLBP layer in the baseline Alexnet and the Alexnet equipped with G-RLBP layer respectively.

Comparing Figure 10a,b, it is worth noticing that, when the input image is injected with some noise, some random noise points appear in the feature maps of the pooling layer. Meanwhile, the crucial edges of the output are no longer clear, and some of the original texture information is even lost in some feature maps. The noise in the image can severely affect the output of some intermediate layers in the networks, and the noise accumulates layer by layer which would eventually reduce the recognition rate of the whole network. However, once the network is equipped with the G-RLBP pooling layer, as Figure 10c shows, the noise injected into the output feature maps can be effectively decreased, and some edges in the feature maps of the G-RLBP also become much clearer compared with Figure 10b.

In Table 3 and Table 4, we summarize the recognition rates when the testing images were injected with Gaussian noise and salt and pepper noise respectively.

The results in Table 3 and Table 4 show that the recognition rates of the six network models are all decreasing along with the noise intensity, especially for the baseline networks with max pooling layer and data augmentation. The fourth and seventh columns in Table 3 and Table 4 indicate the training data of the Alexnet and ZF-5net were injected with different intensity of random noise (Gaussian and salt and pepper noise). When the Gaussian noise intensity increases to $\sigma^{2}=0.005$, the recognition rates of the baseline networks with max pooling layer begin to be lower than 50%. The original pooling method is sensitive to noise, which brings about poor performance when the input images are affected by slight noise. In the tables, we can see that, when the training data are augmented with random noise, the recognition results are much better than the original networks with max pooling method. Furthermore, in comparison, the G-RLBP pooling method proposed in this paper gets the best results, and it is robust to noise. Although the recognition rates of the two networks equipped with the G-RLBP pooling layer are also decreasing along with the noise intensity, the recognition results in these cases are much better than those of the baseline networks. Even if the intensity of the Gaussian noise reaches $\sigma^{2}=0.01$, the recognition rates are also higher than 50%, which indicates that the G-RLBP pooling layer is more effective than the original. The main reason is that, when the networks are equipped with the G-RLBP layer, a smaller weight would be assigned to the pixel that is likely affected by noise. Thus, after the G-RLBP pooling, the noise injected into the feature maps would be kept within a smaller extent. In addition, when the testing images are clean ($\sigma^{2}=0$ in Table 3 or *d* = 0 in Table 4), the recognition rates of the networks with G-RLBP are also slightly higher than those of the baseline networks. In conclusion, the G-RLBP can be used to reduce the model dimensions as the original pooling method and lower the noise interference to the networks in the real-world applications simultaneously.

Finally, we also evaluated the time cost of these network models both at the stage of training and classification, as Table 5 shows. All the network models in our experiments are trained 80 epochs. The classification times in Table 5 only refer to the 4096-feature extraction times here since the feature matching times of all the network models are the same.

In Table 5, we can see that it is difficult to train a network with Data Augmentation, since it has the maximum training data. Compared with the network with a max pooling layer, the classification time of the G-RLBP pooling method is a little longer. However, considering the improvement of the G-RLBP pooling method on the recognition rates in Table 3 and Table 4, the weakness on the classification time-consuming is acceptable.

### 3.3. Experiments on the AR Database

The second set of experiments were conducted on the AR database which contains 126 subjects with 13 images for each subject. The experimental settings were the same as Experiment 1. We chose one image from each subject as the training set per turn. Finally, all recognition rates were averaged as the final results. The AR database is more challenging than the ORL database with more subjects and some uncontrolled conditions.

**Figure 11:**
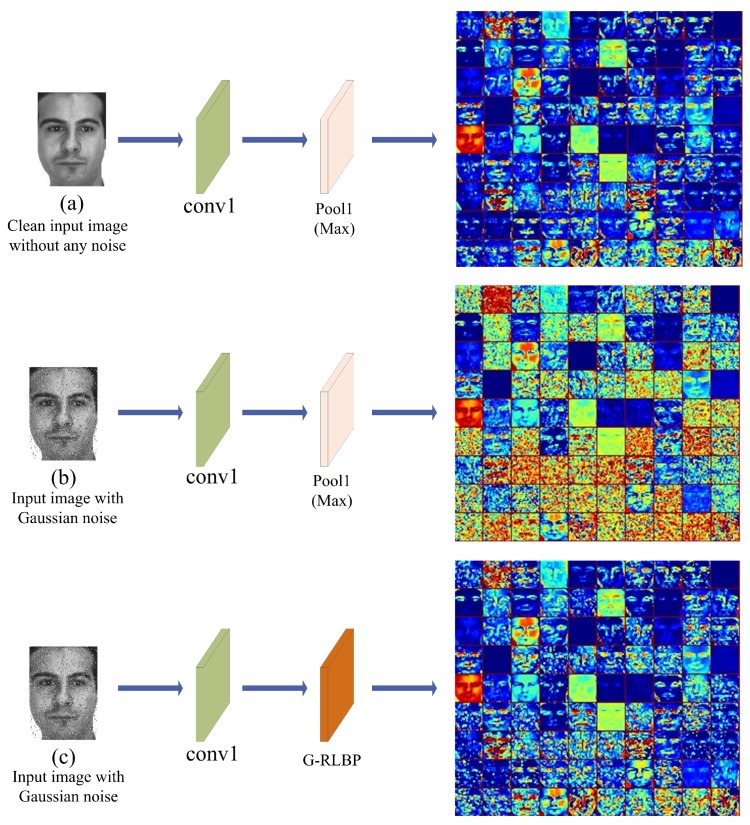
The feature maps of different pooling layers in the Alexnet. Conv1 indicates the first convolutional layer in the networks. (**a**) The output of Pool1 layer without injecting any noise into the input image in the baseline Alexnet; (**b**) the output of Pool1 layer in the baseline Alexnet when the input image is injected with Gaussian noise ($\sigma^{2}=0.005$); and (**c**) the output of the G-RLBP pooling layer when the input image is injected with the same noise as (**b**).

A face image in the AR database is selected randomly, and the image was fed into the networks (Figure 10). The visual results are shown in Figure 11.

It is evident that the edges and other texture information are severely affected in Figure 11b by noise since the max pooling method has no response to noise containing in the inputs. Thus, many random noise points mixed in the feature maps are preserved after pooling. If the max pooling layer is replaced by the G-RLBP pooling method, we can see clearly that the noise impact is lowered effectively (Figure 11c). Furthermore, some crucial texture edges in Figure 11c become more recognizable compared with Figure 11b.

We also quantitatively analyzed the recognition rates of these network models (Table 6 and Table 7) when the testing images were injected with different intensity of Gaussian noise and salt and pepper noise. The recognition rates of the six network models in this section are much lower than those of Experiment 1. If the testing images are clean, the networks succeed to make a nearly 2% improvement of the recognition rates when the networks are equipped with the proposed G-RLBP pooling layer compared with the max pooling layer. The recognition rates of the six network models in Table 6 and Table 7 begin to decrease along with the intensity of noise injected into the testing images, especially in the four baseline networks with max pooling layer and data augmentation. However, the networks with G-RLBP have better performance than the baseline networks. This is mainly because the G-RLBP pooling method is less sensitive to noise and can preserve more crucial texture information of the input feature maps, thus further gets more discriminative features. However, if testing images are severely affected by noise, the six network models are all unable to get good performance, it is even difficult for a human to identify the contour information of the images in this case.

### 3.4. Experiments Based on the GoogleNet

GoogleNet is another effective visual recognition model which has been used in many recognition fields such face recognition, image classification, target tracking and so on. Here, we conduct some experiments on this model to further evaluate our pooling layer. We use the entire CASIA-WebFace database to train our models. For data augmentation, the training data were injected with different intensity of Gaussian noise. The others experimental settings were the same as Experiments 1 and 2. The testing data were chosen from the ORL database and the AR database, respectively. Before the images were fed into the networks, they were injected with different intensity of Gaussian noise. Table 8 and Table 9 show the results. Finally, to make the experimental results more convincing, we also utilized the average filter and the BM3D algorithm [30] as pre-processing steps, respectively, to remove the noise injected in the testing data, as can be seen in the fourth and fifth columns of Table 8 and Table 9.

We can see that the average filter and the BM3D algorithm are both effective when there is some slight noise injected in the testing images ($\sigma^{2}=0.002$ in Table 8 and Table 9). Comparing with the average filter, the BM3D algorithm is more robust with better recognition performance than the average filter in this case. However, when the testing images are clean, the recognition rates of the average filter and the BM3D algorithm are both reduced to some extent. Nevertheless, the G-RLBP pooling method, which gets the best recognition results in all cases, sometimes still has excellent performance even when the testing images are seriously affected by noise. It is also clear that, when the G-RLBP pooling method is transferred to other networks such as the GoogleNet, it can also improve the recognition performance. Thus, the proposed G-RLBP pooling method is effective and can be used in more modern CNN architectures.

## 4. Discussion

In this paper, we propose the G-RLBP pooling method to down sample the feature maps of convolutional layers. Our work has two main contributions: (1) With the robust LBP guiding, each pixel in the input feature maps is assigned with a different weight based on the probability affected by noise. In this way, the proposed G-RLBP can successfully remove the pixels which are likely to be affected by noise and then calculate the weighted average of the rest pixels as the final results to get more noise-robust features. (2) The proposed pooling method can extract more discriminative information from the feature maps and preserve more crucial edges of the face images during the down-sampling. The experimental results in Section 3 show that the proposed G-RLBP pooling method can be used as an effective method to further improve the performance of deep CNNs. It gets the best recognition results comparing with the max pooling method and data augmentation by injecting different random noise into the training data. Especially, in some uncontrolled noisy conditions, the networks equipped with the G-RLBP pooling layer can get better performance. It would be our future work to improve further our networks to adapt to some more complex conditions, such as performing experiments based on some other modern CNN architectures using different image databases with varying degrees of challenge, and so on. It should be mentioned that the proposed G-RLBP pooling method can only be used in the face recognition field at present. Thus, transferring this method to other fields would be our main work in the future. Another possible future work is to involve sparsity-based models [31] to further improve cost-effectiveness and robustness of the recognition system.

References

## Figures and Tables

**Table 1:** The recognition rates (%) of the Alexnet and ZF-5net with different intensity of Gaussian noise.

	$\sigma^{2}=0$	$\sigma^{2}=0.002$	$\sigma^{2}=0.005$	$\sigma^{2}=0.01$
Alexnet	81.16	73.48	64.13	46.72
ZF-5net	81.24	75.62	67.50	49.07

**Table 2:** The configurations of the Alexnet and ZF-5net.

Model	Alexnet		ZF-5net	
	Filter Size/Stride	Output Size	Filter Size/Stride	Output Size
Conv1	11×11/4	96×55×55	7×7/2	96×111×111
Pool1	3×3/2	96×27×27	3×3/2	96×55×55
Conv2	5×5/1	256×27×27	5×5/1	256×55×55
Pool2	3×3/2	256×13×13	3×3/2	256×27×27
Conv3	3×3/1	384×13×13	3×3/2	512×13×13
Conv4	3×3/1	384×13×13	3×3/1	1024×13×13
Conv5	3×3/1	256×13×13	3×3/1	512×13×13
Pool5	3×3/2	256×6×6	3×3/2	256×6×6
Fc5	-	4096	-	4096
Fc6	-	4096	-	4096
Fc7	-	100	-	100

**Table 3:** The recognition rates (%) of different pooling methods and data augmentation based on the Alexnet and ZF-5net on the ORL database with different Gaussian noise.

		Alexnet+ Max	Alexnet+ Data Aug.	Alexnet+ G-RLBP	ZF-5net+ Max	ZF-5net+ Data Aug.	ZF-5net+ G-RLBP
$\sigma^{2}=0$	Chi Square	85.38	85.67	86.42	84.12	85.88	**87.68**
	Euclidean	83.06	83.87	**85.25**	80.00	81.64	83.69
	Cosine	89.44	90.12	**92.74**	85.33	86.56	87.72
$\sigma^{2}=0.002$	Chi Square	65.15	76.43	80.55	69.44	77.75	**81.42**
	Euclidean	61.32	80.32	**85.34**	66.11	78.47	82.25
	Cosine	60.56	78.54	82.51	68.06	79.21	**83.50**
$\sigma^{2}=0.005$	Chi Square	47.22	58.84	72.47	52.48	60.22	**75.48**
	Euclidean	36.94	55.32	69.15	48.62	56.65	**72.45**
	Cosine	48.89	56.76	70.54	53.87	57.80	**73.66**
$\sigma^{2}=0.01$	Chi Square	23.61	26.22	54.53	38.89	40.34	**59.41**
	Euclidean	15.83	20.11	49.87	29.44	32.90	**51.03**
	Cosine	20.87	23.87	52.32	36.72	36.45	**57.15**

**Table 4:** The recognition rates (%) of different pooling methods and data augmentation based on the Alexnet and ZF-5net on the ORL database with different salt and pepper noise.

		Alexnet+ Max	Alexnet+ Data Aug.	Alexnet+ G-RLBP	ZF-5net+ Max	ZF-5net+ Data Aug.	ZF-5net+ G-RLBP
d=0	Chi Square	85.38	85.77	86.42	84.12	85.53	**87.68**
	Euclidean	83.06	84.46	**85.25**	80.00	81.24	83.69
	Cosine	89.44	91.20	**92.74**	85.33	86.43	87.72
d=0.05	Chi Square	37.78	59.72	65.65	45.78	60.21	**68.55**
	Euclidean	35.62	56.44	62.85	41.11	57.97	**63.32**
	Cosine	35.98	57.83	**64.72**	45.78	59.44	64.58
d=0.1	Chi Square	24.56	34.21	42.15	30.44	37.83	**48.87**
	Euclidean	19.86	27.84	39.42	23.55	30.22	**42.84**
	Cosine	21.67	30.76	**45.57**	28.56	35.17	42.05
d=0.15	Chi Square	19.05	22.13	32.12	24.72	26.43	**33.65**
	Euclidean	15.72	18.04	**25.96**	18.85	21.36	24.17
	Cosine	17.39	20.42	**29.98**	20.24	18.12	21.10

**Table 5:** The training and classification time of different pooling methods and data augmentation.

	Alexnet+ Max	Alexnet+ Data Aug.	Alexnet+ GRLBP	ZF-5net+ Max	ZF-5net+ Data Aug.	ZF-5net+ GRLBP
training (h)	26	50	32	31	63	40
classification per image (ms)	26.983	27.021	30.478	27.225	27.219	30.694

**Table 6:** The recognition rates (%) of different pooling methods and data augmentation based on the Alexnet and ZF-5net on the AR database with different Gaussian noise.

		Alexnet+ Max	Alexnet+ Data Aug.	Alexnet+ G-RLBP	ZF-5net+ Max	ZF-5net+ Data Aug.	ZF-5net+ G-RLBP
$\sigma^{2}=0$	Chi Square	67.97	66.74	65.85	66.41	67.56	**68.52**
	Euclidean	65.77	66.12	67.84	68.02	68.27	**69.88**
	Cosine	64.92	65.96	69.44	70.38	71.23	**72.52**
$\sigma^{2}=0.002$	Chi Square	34.42	42.72	53.86	39.69	45.51	**57.74**
	Euclidean	30.54	40.11	50.65	36.87	42.28	**52.42**
	Cosine	31.17	41.54	51.23	40.59	46.73	**55.45**
$\sigma^{2}=0.005$	Chi Square	22.87	28.41	44.12	26.72	30.07	**47.41**
	Euclidean	19.43	26.76	42.25	22.54	28.11	**42.75**
	Cosine	24.51	28.83	**46.36**	25.06	27.43	45.25
$\sigma^{2}=0.01$	Chi Square	10.02	16.12	30.22	12.26	17.34	**33.12**
	Euclidean	9.63	15.03	29.85	10.24	16.12	**34.58**
	Cosine	12.52	17.43	31.89	15.58	18.11	**36.64**

**Table 7:** The recognition rates (%) of different pooling methods and data augmentation based on the Alexnet and ZF-5net on the AR database with different salt and pepper noise.

		Alexnet+ Max	Alexnet+ Data Aug.	Alexnet+ G-RLBP	ZF-5net+ Max	ZF-5net+ Data Aug.	ZF-5net+ G-RLBP
d=0	Chi Square	67.97	65.79	65.85	66.41	67.41	**68.52**
	Euclidean	65.77	66.83	67.84	68.02	68.12	**69.88**
	Cosine	64.92	66.32	69.44	70.38	71.44	**72.52**
d=0.05	Chi Square	30.02	36.42	44.51	34.69	40.01	**49.42**
	Euclidean	28.85	36.11	40.14	30.05	38.83	**48.33**
	Cosine	31.58	37.23	42.28	33.67	39.97	**48.87**
d=0.1	Chi Square	21.87	27.54	39.96	21.43	28.82	**43.22**
	Euclidean	20.63	26.63	36.58	19.85	25.44	**38.16**
	Cosine	23.38	29.36	38.74	22.36	27.87	**39.55**
d=0.15	Chi Square	9.67	14.11	**22.13**	11.48	14.92	20.65
	Euclidean	5.96	12.30	**18.85**	8.82	12.14	17.52
	Cosine	11.20	13.04	19.63	10.20	13.49	**21.34**

**Table 8:** The recognition rates (%) of different pooling methods, the average filter, the BM3D algorithm, and data augmentation based on the GoogleNet on the ORL database with different Gaussian noise.

		GoogleNet+ Max	GoogleNet+ Ave. Filter	GoogleNet+ BM3D	GoogleNet+ Data Aug.	GoogleNet+ GRLBP
$\sigma^{2}=0$	Chi Square	88.51	87.24	88.43	90.12	**92.54**
	Euclidean	86.32	85.37	87.57	89.45	**92.33**
	Cosine	89.76	88.16	89.21	91.12	**93.69**
$\sigma^{2}=0.002$	Chi Square	50.27	57.46	87.28	60.63	**87.47**
	Euclidean	48.12	56.10	**86.14**	59.84	85.63
	Cosine	51.48	57.23	87.02	62.01	**87.76**
$\sigma^{2}=0.005$	Chi Square	38.65	44.76	61.07	47.21	**71.22**
	Euclidean	38.04	43.02	59.11	46.33	**70.91**
	Cosine	39.87	45.93	61.94	48.88	**72.51**
$\sigma^{2}=0.01$	Chi Square	18.59	20.12	40.46	23.14	**50.14**
	Euclidean	16.49	19.43	39.17	22.07	**48.35**
	Cosine	19.06	21.56	40.89	24.78	**51.55**

**Table 9:** The recognition rates (%) of different pooling methods, the average filter, the BM3D algorithm, and data augmentation based on the GoogleNet on the AR database with different Gaussian noise.

		GoogleNet+ Max	GoogleNet+ Ave. Filter	GoogleNet+ BM3D	GoogleNet+ Data Aug.	GoogleNet+ GRLBP
$\sigma^{2}=0$	Chi Square	70.22	69.15	69.34	72.10	**74.87**
	Euclidean	67.89	67.23	69.21	70.03	**73.46**
	Cosine	71.83	69.47	70.07	73.14	**76.17**
$\sigma^{2}=0.002$	Chi Square	39.88	46.14	69.12	50.64	**70.78**
	Euclidean	36.56	45.28	69.09	48.73	**70.21**
	Cosine	40.14	46.93	70.42	50.26	**71.38**
$\sigma^{2}=0.005$	Chi Square	25.11	30.57	46.74	36.65	**57.98**
	Euclidean	25.52	29.42	46.22	35.49	**57.35**
	Cosine	26.77	31.61	47.91	37.82	**59.01**
$\sigma^{2}=0.01$	Chi Square	14.40	16.28	30.03	18.17	**42.74**
	Euclidean	13.29	14.93	29.83	17.50	**40.37**
	Cosine	15.32	16.11	31.14	19.59	**43.83**
